# Pre-Treatment Deep Curettage Can Significantly Reduce Tumour Thickness in Thick Basal Cell Carcinoma While Maintaining a Favourable Cosmetic Outcome When Used in Combination with Topical Photodynamic Therapy

**DOI:** 10.1155/2011/240340

**Published:** 2011-11-15

**Authors:** Eidi Christensen, Cato Mørk, Olav Andreas Foss

**Affiliations:** ^1^Department of Dermatology, St. Olav's University Hospital HF, Institute of Cancer Research and Molecular Medicine, Faculty of Medicine, Norwegian University of Science and Technology (NTNU), 7030 Trondheim, Norway; ^2^Dermatology Unit, Institute of Cancer Research and Molecular Medicine, Faculty of Medicine, Norwegian University of Science and Technology (NTNU), 7030 Trondheim, Norway; ^3^Department of Orthopaedics, St. Olav's University Hospital HF and Department of Neuroscience, Faculty of Medicine, Norwegian University of Science and Technology (NTNU), 7030 Trondheim, Norway

## Abstract

Topical photodynamic therapy (PDT) has limitations in the treatment of thick skin tumours. The aim of the study was to evaluate the effect of pre-PDT deep curettage on tumour thickness in thick (≥2 mm) basal cell carcinoma (BCC). Additionally, 3-month treatment outcome and change of tumour thickness from diagnosis to treatment were investigated. At diagnosis, mean tumour thickness was 2.3 mm (range 2.0–4.0). Pre- and post-curettage biopsies were taken from each tumour prior to PDT. Of 32 verified BCCs, tumour thickness was reduced by 50% after deep curettage (*P* ≤ 0.001). Mean tumour thickness was also reduced from diagnosis to treatment. At 3-month followup, complete tumour response was found in 93% and the cosmetic outcome was rated excellent or good in 100% of cases. In conclusion, deep curettage significantly reduces BCC thickness and may with topical PDT provide a favourable clinical and cosmetic short-term outcome.

## 1. Introduction

Basal cell carcinoma (BCC) is the most common cancer in the white population, and its incidence is still increasing [1, 2]. This is a slow-growing, locally invasive epidermal skin tumour that can cause considerable patient morbidity [3, 4]. BCC most often arises on sun exposed, cosmetic sensitive skin areas such as the face [4].

Among several therapeutic options available for the treatment of this tumour excision surgery is regarded as the most effective [5]. However, not all patients are qualified for surgery. Excision surgery may be challenging in certain anatomic areas, cause cosmetic disfigurement, or result in complications like scar formation and functional impairment [6]. 

Topical PDT, with beneficial cosmesis, may in such cases be an attractive treatment option [7, 8]. This method involves the activation of a topically applied photosensitizer by light in the presence of tissue oxygen, starting a photochemical reaction in the targeted cells [9].

Five-year clearance rates in BCC from 64 to 81% are reported [10–13].

Evidence-based guidelines support the use of topical PDT in the treatment of BCC, particularly low risk, superficial lesions [14, 15]. A challenge is the limited penetration of the photosensitizing agents down to about 1.0 to 2.0 mm depth [16–18] and also limitation of red light to penetrate the skin [9]. The treatment efficacy in BCC with thickness ≥2.0 mm may therefore be reduced. 

Among several strategies to increase PDT effect, pre-treatment curettage has been shown to improve treatment efficacy in nodular tumours [19]. The combination of curettage ahead of PDT is today commonly used, even though data to supports its effect is rare [19, 20]. 

It is recommended to perform a pre-treatment biopsy to obtain an indication of tumour thickness [21]. However, the essential question from a clinical point of view is how thick the BCC appears after curettage. 

Consequently, it is of interest to examine to what extent tumour thickness may be reduced by deep curettage and examine to which degree this may affect treatment outcome.

The main objective of this study was to evaluate the effect of deep curettage on tumour thickness in thick BCC. Additionally, short-term treatment efficacy and cosmesis as well as changes in tumour thickness from diagnosis to treatment were investigated.

## 2. Material and Method

The study was conducted at the Department of Dermatology, St. Olav's Hospital HF, Trondheim over a two-year period. Patients with histological verified BCC ≥2.0 mm thick, selected for PDT were eligible. The study was approved by the Regional Ethics Committee, and informed consent was obtained from all patients before study entry.

The size was defined as the mean of the length and width of the lesion. Pre- and post-curettage biopsies were taken from the central tumour area by one investigator (EC) using a 2.0–3.0 mm disposable punch biopsy (Stiefel Laboratories Ltd., Sligo, Ireland). The centre was defined as the midpoint of the line following the greatest tumour length and was marked using a skin marker. The biopsies were taken approximately 0.5 mm from either side of the midpoint. In lesions with a central ulceration, the biopsies were taken outside of the ulcerated area, along the line following the greatest tumour length, approximately 1.0 mm apart. Bleeding after deep curettage was dried with gauze prior to the taking of post-curettage biopsy.

The biopsy tissue was fixed in 10% formaldehyde, routinely processed, embedded in paraffin, cut perpendicular to the skin surface at three places in sections of 4 *μ*m, and stained with haematoxylin, eosin, and saffron (HES). 

The histological prepared slides were examined by pathologists at St. Olav's University Hospital. The tumour thickness was measured from the stratum corneum to the bottom of the tumour nest. The pre-curettage biopsies were classified histologically as of nonaggressive (nodular) or aggressive (micronodular and morpheform/infiltrative) by one pathologist. 

To ensure as little variation as possible deep curettage was performed by one investigator (EC). The procedure was comprised of an intratumoural debulking within clinical margins of the lesion followed by multiple passes of curettage in various directions across the tumour base. A small surgical curette was used to remove soft, friable tissue and a disposable 4 mm ring curette (Stiefel Laboratories LTD, Sligo, Ireland) used to remove hyperkeratosis and crusts and to scrape clinically firm tumour areas. In addition, a 4 mm broad brim of normal appearing skin surrounding the tumour was superficially scraped using a ring curette only to remove stratum corneum.

The area was then treated with PDT using methyl aminolevulinate (MAL) as a precursor of photosensitive porphyrins. MAL cream (Metvix, Galderma, France) was applied onto the treatment site in a 1 mm thick layer and occluded with a light-shielding dressing. Any residual cream was wiped off after 3 hours and the area exposed to red light (570–670 nm). A noncoherent LED light source (Aktilite, Galderma, France) was used with a fluence rate of 70–100 mW/cm^2^ and light dose of 37 J/cm^2^.

Efficacy was evaluated by dermatologists through inspection and palpation three months after treatment and classified as either in complete response (complete disappearance of tumour) or as noncomplete response.

The cosmetic outcome in the clinical complete response areas was rated on a 4-point ordinal scale as (1) excellent (absence of any stigmata of treatment), (2) good (slightly visible fibrosis, atrophy or change in pigmentation), (3) poor (moderate visible fibrosis, atrophy, or change in pigmentation), or (4) fair (marked visible fibrosis, atrophy or change in pigmentation).

All statistics were calculated using SPSS version 15 (SPSS Inc., Chicago, IL, USA). 

Data distributions were examined by Q-Q plots. Two-tailed, one-sample Student *t*-test was used to analyse the differences in tumour thickness. Simple linear regression was used to analyse the association between treatment waiting time (independent value) and tumour thickness reduction (dependent value). One-way ANOVA was used to analyse possible differences in tumour reduction and cosmetic result. *P* < 0.05 was considered statistically significant.

## 3. Results

A total of 36 patients with each one BCC tumour were included. Twenty-one patients were men, mean age 72 years (range 39–92), and 15 were women, mean age 77 years (range 62–85).

Thirty-four of the tumours were located to scalp or face and two located to the back. Mean lesion size was 11 mm (range 3–38). The mean diagnostic biopsy tumour thickness was 2.3 mm (range 2.0–4.0). The average time from diagnosis to treatment was 91 days (range 13–339). In three cases BCC was not demonstrated in either the pre- or post-curettage biopsies. One post-curettage biopsy was lost after fixation. Thirty-two lesions were included in the analyses. Histologically, 20 tumours were of nonaggressive and 12 were of aggressive (micronodular = 3, morpheaform/infiltrative = 9) growth type. All data were found to be normally distributed.

Mean tumour thickness before curettage was 2.0 mm (range 0.7–4.0) and after curettage was 1.0 mm (range 0.0–3.1). The differences between measurements was statistically significant (Table 1). The difference between pre- and post-curettage thickness measurements for the aggressive and nonaggressive subtypes of BCC were also statistically significant, *P* = 0.001 (Table 1). In three cases the measurements before and after curettage were identical. In one case tumour thickness measurement was 0.1 mm greater after curettage. 

A difference between the diagnostic and pre-curettage mean biopsy measurement of 0.3 mm (SD 0.7) was found to be statistically significant (*P* = 0.04). For nonaggressive tumours only, the difference was 0.34 mm (SD 0.6) (*P* = 0.02) and for the aggressive subtype it was 0.13 mm (SD 0.9) (*P* = 0.60). 

**Figure 1:**
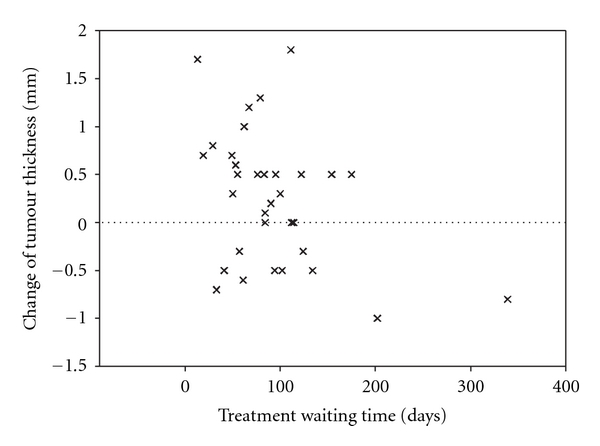
Scatter diagram showing changes of BCC thickness measurements from diagnosis to treatment. Reduction of thickness is indicated by positive sign.

A weak but statistical significant linear regression was found between the treatment waiting time and tumour reduction of the same period (*P* = 0.03, adjusted *r* square 0.13) (Figure 1). One patient was clearly an outlier, waiting more than 300 days before treatment. By excluding this patient from the analyses, the linear regression was no longer statistically significant (*P* = 0.11).

### 3.1. Three-Month Follow-Up

Five of the initial 36 included lesions were missed for 3-month PDT follow-up. Two lesions in two patients were excised after a reassessment of treatment shortly after the first PDT session. This decision was based on the post-curettage biopsy tumour thickness measurement, combined with factors as lesion size and location. Further, two patients discontinued the scheduled PDT sessions because of the experience of severe pain during light exposure. They were referred to treatment by excision surgery. One patient with one lesion died from a non-PDT-related cause.

Data from the five dropout patients were excluded from the data analyses using the per-protocol population (*n* = 31) for the 3-month follow-up. When disregarding the three pre- and post-curettage non-BCC-verified cases, noncomplete response was observed in 2 out of 28 (7%) treated tumours. One was a nodular tumour with a thickness reduction of 0.4 mm and the other was an aggressive subtype showing a 0.1 mm increase after curettage. 

**Figure 2:**
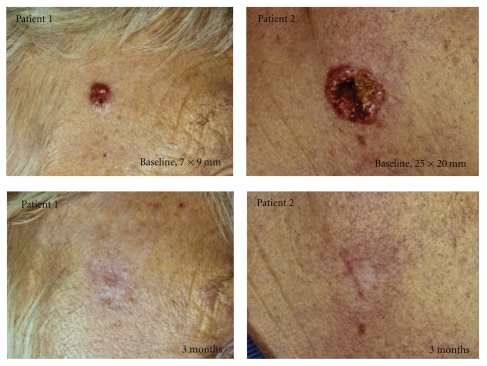
Response to deep curettage and MAL-PDT in thick BCC; patient 1 with a tumour on the temple and patient 2 with a tumour on the lower cheek. Cosmetic outcome was rated as good in both cases.

The cosmetic outcome was rated excellent in 11 of 26 (42%) and good in 15 of 26 (58%) of cases, of which two examples are demonstrated in Figure 2.

No difference between tumour thickness reduction after curettage and the cosmetic outcome was found (*P* = 0.36).

## 4. Discussion

The main finding of this study was a mean 50% reduction of BCC tumour thickness measurement after deep curettage. The reduction was significant for both the aggressive (nodular) and nonaggressive (micronodular and morpheaform/infiltrative) histological subtypes. 

These findings support the practice of pre-treatment curettage as an effective intervention for tumour thickness reduction before the use of PDT in thick BCC.

There are a number of BCCs that are currently difficult to treat by traditional invasive therapies because of size, site, or multiple lesion presentation, particularly if trying to avoid complications such as scar formation. Although treatment by topical PDT has been shown to be less effective compared with excision surgery in nodular BCC [22, 23], cosmetic outcome for PDT is significantly better [22]. 

Despite the use of deep curettage ahead of PDT, we still achieved a favourable cosmetic result assessed as good or excellent in all the evaluated cases. 

The 3-month complete response rate of 93% is comparable to published short-term treatment results after MAL-PDT following curettage showing clearance from 91 to 97% for superficial and from 82 to 91% for nodular BCC [11, 22, 24, 25]. However, in these studies the measurement of tumour thickness was either not stated or was clinically evaluated only. 

Most BCCs appear in the face and neck area [3] of which the nodular and aggressive morpheaform/infiltrating types predominate [26]. Aggressive growth types may penetrate more deeply into the dermis and are often fibrotic [4]. Encouraging, therefore, was the present finding of tumour thickness reduction to be even more pronounced in the aggressive compared to the nonaggressive type after deep curettage.

There are, however, various factors that may have affected the thickness measurements reported in this study. The pre- and post-curettage punch biopsies were, as described, not taken from identical tumour areas within the tumour. BCC can have an irregular growth with infiltrating extensions [27]. The disparity between thickness measurements found in tissue samples taken from different areas of individual BCC tumours has been shown to increase with increased tumour depth [28]. This may explain why tumour thickness in one case showed a 0.1 mm increase and in three other cases showed no reduction after curettage. Another source of inaccuracy may be that sections for histology can “curl up” when placed on the slides, giving rise to abbreviated measurements. Variations of measurement may also be influenced by several pathologists being involved. Nevertheless, despite possible inaccuracies in the measurements, the study results clearly show that deep curettage in a large number of cases greatly reduces BCC tumour thickness.

BCCs grow slowly and may take years to double in size [3]. However, this study demonstrated a significant mean tumour thickness reduction of 0.3 mm when comparing the initial diagnostic biopsy with the pre-curettage biopsy measurements. Spontaneous regression of BCC is recognized [29, 30], and biopsy-induced regression is suggested to occur in 24% of tumours [31]. Local immune responses involving activated T-cells infiltrating regressing BCCs with apoptosis of tumour cells may partly explain this phenomenon [30]. Also, BCC is highly dependent on the surrounding stroma for survival. Wound healing processes including nonspecific inflammatory responses can disrupt the tumour and its stroma and may thus play an important role in tumour regression [31].

Similar mechanisms may explain why BCCs in three cases were not detected in either pre- or post-curettage biopsies and apparently underwent spontaneous clearance. However, we cannot exclude sampling error. 

In the treatment of BCC, curettage is frequently combined with other modalities such as surgery, electrodessication, cryosurgery, and topical PDT. It is commonly performed to delineate tumour margins and/or to reduce tumour thickness. To what extent curettage exerts an independent effect on treatment outcome is unclear. 

A few studies report on the treatment of small (≤15 mm) tumours and/or selected groups of BCC with curettage alone [32, 33]. This appears to be effective with long-term recurrence rates shown from 8 to 14%, which is comparable to non-Mohs' standard therapies. In a fairly recent retrospective study a cure rate of 96% including a favourable cosmetic outcome was demonstrated [34]. However, historic controls and/or no specification of tumour thickness limit the data in these studies.

The belief that curettage alone is not sufficient to erase all parts of tumour is, on the other hand, supported by a finding in the study by Jih et al. [35] evaluating the ability of curettage to selectively remove nonmelanoma skin cancer including BCC. Overall, the curette left no residual tumour at the surgical margins in only 12% of cases.

PDT as a topical monotherapy still appears to be a less attractive option for the treatment of thicker BCC. The combination of PDT and pre-treatment curettage has proved more effective compared to treatment by placebo cream and pre-treatment curettage alone. In a randomized, double blinded study of nodular BCC the complete response rates were 73% versus 27%, respectively [36]. 

In a study by Fantini et al. [37], a low response rate of 33% was reported for nodular BCC after treatment with PDT after removal of only scales and crusts from the surface. 

Further, prior debulking curettage to PDT achieved a complete response rate for nodular BCC of 92% compared to non complete response in the control groups [20]. The controls comprised small numbers of tumours treated with PDT only and with curettage alone. 

These results imply that pre-treatment curettage contribute to make nodular BCC more responsive to topical PDT.

Within the PDT regime, pre-treatment curettage is a practical, technique-dependant supportive method practised with great variability. In trying to obtain uniformity of this procedure, the same investigator performed the curettage in all cases in the present study. However, it was difficult to achieve a standardization of the practical exercise, which again may have influenced the study results. 

The main purpose of deep curettage was to erase the main bulk of the tumour within its clinical margins. The purpose was not to remove all parts of the tumour in an attempt to prevent damage afflicted to surrounding tissue, as reflected by the favourable post-treatment cosmetic results obtained.

## 5. Conclusion

The study showed a significant reduction of tumour thickness in thick BCC after deep curettage. A favourable short-term efficacy was found, and cosmesis was maintained following deep curettage and PDT. 

Topical PDT combined with deep curettage may be considered as a treatment option of selected thick BCCs in cases where surgery or other invasive treatment methods are regarded as suboptimal.

##  Source of the Work 

Out-patient clinic at the Department of Dermatology, St. Olav's University Hospital HF, Trondheim, Norway.

##  Conflict of Interests

The authors declare no conflict of interests.

## Figures and Tables

**Table 1:** Mean BCC tumour thickness measurements, before and after curettage.

	Before (mm) (SD)	After (mm) (SD)	Difference (mm) (95% CI)	*P*-value
BCC tumours included *n* = 32	2.0 (0.8)	1.0 (0.8)	1.0 (0.7,1.3)	*P* < 0.001
Nonaggressive type *n* = 20	1.9 (0.7)	1.0 (0.7)	0.9 (0.5,1.2)	*P* = 0.001
Aggressive type *n* = 12	2.3 (0.8)	1.1 (1.0)	1.2 (0.6,1.8)	*P* < 0.001
